# Prevalence of low bone mineral density in robotic-assisted TKA candidates: insights from quantitative CT analysis

**DOI:** 10.1051/sicotj/2025048

**Published:** 2025-10-31

**Authors:** Riccardo Garibaldi, Sébastien Lustig, Martinique Vella-Baldacchino, Paolo Ivan Fiore, Cécile Batailler

**Affiliations:** 1 Orthopaedics Surgery and Sports Medicine Department, FIFA Medical Center of Excellence, Croix-Rousse Hospital, Lyon University Hospital 103 Grande Rue de la Croix-Rousse 69004 Lyon France; 2 Univ Lyon, Claude Bernard Lyon 1 University, IFSTTAR, LBMC UMR_T9406 69622 Lyon France; 3 Department of Orthopedic Surgery and Traumatology, Cantonal Hospital Fribourg, University of Fribourg Chemin des Pensionnats 2–6 1708 Fribourg Switzerland; 4 Musculoskeletal Lab, Imperial College London, South Kensington Campus London SW7 2AZ UK; 5 Service of Orthopaedics and Traumatology, Department of Surgery, Ente Ospedaliero Cantonale (EOC) Via Tesserete 46 6900 Lugano Switzerland

**Keywords:** Osteoporosis, Bone mineral density, Robotic-assisted total knee arthroplasty, Quantitative computed tomography, Opportunistic screening

## Abstract

*Introduction*: Osteoporosis is a prevalent and often underdiagnosed condition that significantly increases the risk of fragility fractures. Dual-energy X-ray absorptiometry (DXA) is the standard diagnostic tool; however, many patients remain unscreened. Preoperative computed tomography (CT) scans obtained for robotic-assisted total knee arthroplasty (TKA) planning present an opportunity for opportunistic osteoporosis screening without additional radiation exposure. *Methods*: A retrospective observational study was conducted on 637 patients (307 males, 330 females) who underwent robotic-assisted TKA between January 2023 and December 2024. Preoperative CT scans were analyzed using quantitative computed tomography (QCT) software to determine *T*-scores, *Z*-scores, and percentage of bone mineral density (BMD) relative to a young-adult reference. Patients were categorized as normal (*T*-score ≥ −1.0), osteopenic (−2.5 < *T*-score < −1.0), or osteoporotic (*T*-score ≤ −2.5). *Results*: Among 597 patients with available *T*-score data, 41.0% were classified as normal, 32.3% as osteopenic, and 26.6% as osteoporotic. Notably, 37.0% of female patients were osteoporotic compared to 15.3% of male patients. Bone density parameters declined progressively with age, with females over 80 years exhibiting a mean *T*-score of −2.53 and BMD at 68.25% of the young-adult reference. *Discussion*: Opportunistic screening using preoperative CT scans in robotic-assisted TKA patients reveals a high prevalence of undiagnosed low BMD, particularly among elderly women. Integrating QCT analysis into the preoperative workflow may facilitate early identification of at-risk individuals, informing surgical planning and enabling timely interventions to improve bone health.

## Introduction

Osteoporosis is a prevalent and often asymptomatic skeletal disorder characterized by decreased bone mineral density (BMD) and microarchitectural deterioration, leading to increased bone fragility and susceptibility to fractures, particularly in the spine, hip, and wrist. It affects nearly 50% of women and up to 20% of men over the age of 50, posing a significant public health challenge in aging populations worldwide [1]. Despite the availability of effective diagnostic and therapeutic interventions, osteoporosis remains substantially underdiagnosed and undertreated. Dual-energy X-ray absorptiometry (DXA) is the current gold standard for assessing BMD, as defined by the World Health Organization (WHO) [2]. However, screening coverage is suboptimal, and a considerable proportion of fragility fractures occur in individuals who have never undergone DXA evaluation [3, 4].

In response, alternative strategies for identifying individuals at risk are being explored. Opportunistic screening using computed tomography (CT) has gained attention, wherein Hounsfield unit (HU) measurements from standard CT images – originally acquired for other clinical indications – serve as surrogate markers for BMD. This approach has demonstrated strong correlations with DXA-derived BMD values across various anatomical sites [5–9]. Notably, it requires no additional radiation exposure, time, or cost, facilitating seamless integration into routine clinical workflows. Several studies have validated the use of HU measurements for opportunistic osteoporosis screening in contexts such as trauma imaging [6], CT colonography [5], spinal surgery [9], and general abdominal CT scans [7, 8]. The proximal femur and lumbar spine are particularly amenable to this method due to their consistent anatomy and relevance in fracture risk assessment [6, 9].

In orthopedic surgery, preoperative knowledge of bone quality can significantly influence decisions regarding implant selection, fixation techniques, and postoperative management. CT scans obtained for robotic-assisted total knee arthroplasty (TKA) planning are highly standardized and technically suitable for bone assessment using HU metrics [10–13]. This presents potentially a valuable yet underutilized opportunity for osteoporosis screening. To our knowledge, no prior studies have investigated the feasibility of opportunistic osteoporosis screening using CT data from TKA planning. Given the high prevalence of osteoporosis among patients undergoing joint replacement, we hypothesize that opportunistic CT scans performed for robotic TKA could help identify a significant number of patients with severe osteopenia. This approach could offer significant clinical benefits by identifying at-risk individuals and informing both surgical and medical management strategies.

## Material and methods

### Study design and population

This retrospective observational study included consecutive patients who underwent robotic-assisted TKA at Hôpital de la Croix-Rousse, Lyon, France, between January 1, 2023, and December 31, 2024. All procedures were planned using the Mako SmartRobotics^TM^ System (Stryker, USA), which necessitates a preoperative CT scan.

A total of 636 patients (307 males and 329 females) were initially considered. In cases where a proximal femoral prosthesis or osteosynthesis material was present on the ipsilateral side, the contralateral hip was used for analysis. Patients were excluded from quantitative analysis if the contralateral hip was not adequately visualized in the CT scan (*n* = 39).

### Imaging protocol and bone mineral density assessment

**Figure 1:**
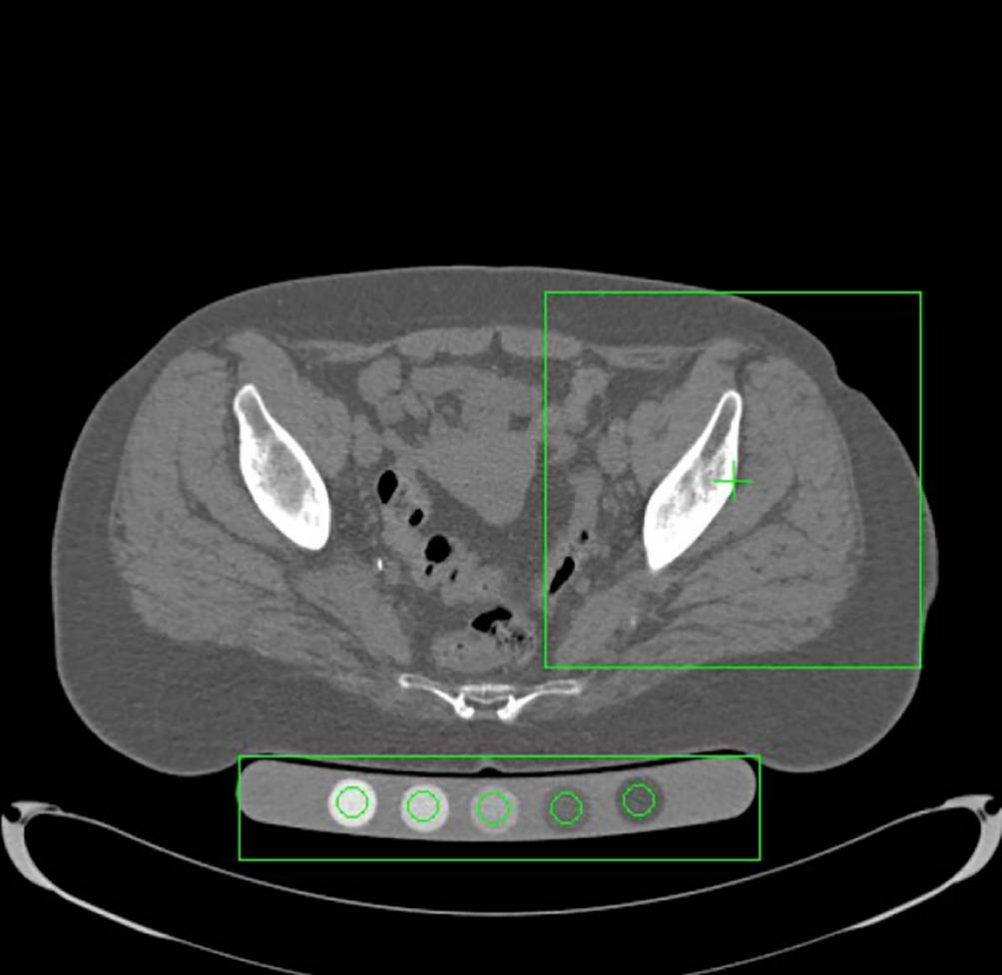
Automatic selection of regions of interest (ROIs) for QCT analysis. Axial CT slice processed with the CTXA Hip module (QCT Pro, Mindways). The green boxes indicate the automated placement of the hip ROI and the calibration phantom, which are used to extract bone mineral density values for osteoporosis assessment.

**Figure 2:**
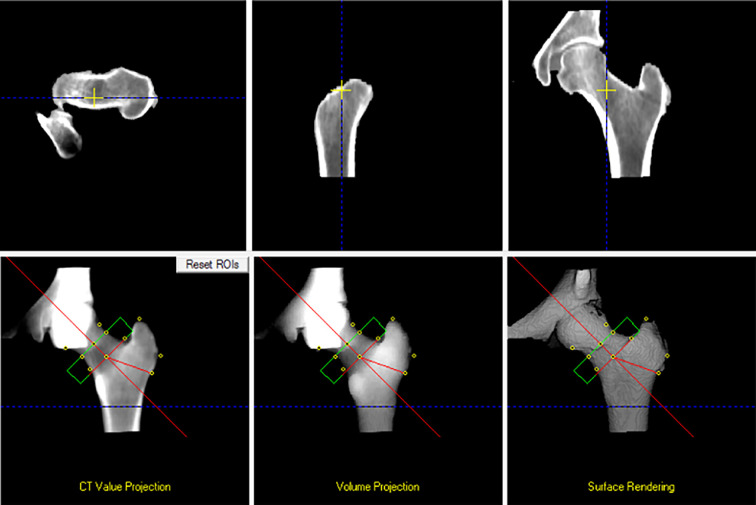
3D reconstruction and ROI placement for bone mineral density measurement. Multiplanar views (CT value projection, volume projection, and 3D surface rendering) showing the automatic placement of standard ROIs (femoral neck, intertrochanteric, and total hip) used for calculating *T*-score and %BMD according to ISCD guidelines.

Preoperative CT scans were acquired as part of the standard robotic TKA workflow. The scans were exported and processed using the QCT Pro – CTXA HIP Module (Mindways Software Inc., Austin, TX, USA), a validated software for opportunistic bone density analysis based on quantitative computed tomography (QCT) (Figures 1 and 2).

The analysis included measurements of:

- *T*-score;
- *Z*-score;
- Bone mineral density (BMD) expressed as a percentage of the reference young-adult mean (%BMD).

These values were automatically computed in the integral region of interest (ROI) encompassing the femoral neck and intertrochanteric region, following the standards set by the International Society for Clinical Densitometry (ISCD).

Patients were categorized based on WHO criteria:

- Normal: *T*-score ≥ −1.0;
- Osteopenic: −2.5 < *T*-score < −1.0;
- Osteoporotic: *T*-score ≤ −2.5.

### Data collection

Demographic and clinical data, including age and sex, were extracted from institutional records. No additional radiation exposure or procedures were performed beyond routine clinical care.

### Statistical analysis

Descriptive statistics were used to evaluate *T*-score, *Z*-score, and %BMD, stratified by sex and age groups. In addition, multivariate linear regression was performed to assess the association between age, sex, and the *Z*-score. Logistic regression was also conducted to evaluate the effect of sex on the likelihood of osteoporosis, osteopenia, and normal bone mass. Statistical significance was defined as *p* < 0.05. Odds ratios (OR) and 95% confidence intervals (CI) were calculated for categorical outcomes..

## Results

A total of 636 patients underwent robotic-assisted TKA between January 1, 2023, and December 31, 2024. After excluding 39 patients due to inadequate visualization of the contralateral hip on preoperative CT scans, 597 patients (307 males [48.2%], 330 females [51.8%]) were included in the final analysis. The mean age of the cohort was 68.4 ± 8.7 years, with an age range between 45 and 89 years (Table 1).

**Table 1:** General characteristics.

Parameter	Total	Males	Females
Number of patients	637	307 (48.2%)	330 (51.8%)
*T*-score (mean)	−1.60	−1.05	−2.06
*Z*-score (mean)	−0.20	+0.24	−0.59
%BMD (mean)	79.65%	86.51%	73.79%

Quantitative computed tomography (QCT) analysis showed that the mean *T*-score for the entire cohort was −1.60 ± 1.10, with a mean *Z*-score of −0.20 ± 0.95, and a mean %BMD of 79.65% ± 12.4%. When stratified by sex, male patients exhibited a mean *T*-score of −1.05 ± 0.95, a mean *Z*-score of +0.24 ± 0.85, and a %BMD of 86.51% ± 10.2%. Female patients showed significantly lower values, with a mean *T*-score of –2.06 ± 1.05, a mean *Z*-score of –0.59 ± 0.90, and a %BMD of 73.79% ± 11.5%.

According to the WHO classification, 245 patients (41.0%) were classified as having normal bone mass (*T*-score ≥ –1.0), 193 patients (32.3%) were classified as osteopenic (*T*-score between –2.5 and –1.0), and 159 patients (26.6%) were classified as osteoporotic (*T*-score ≤ –2.5). (Table 2) The sex-specific distribution revealed a higher prevalence of osteoporosis among female patients, with 84 females (25.5%) being classified as normal, 124 (37.6%) as osteopenic, and 122 (37.0%) as osteoporotic. In contrast, among males, 178 (58.0%) were normal, 82 (26.7%) were osteopenic, and 47 (15.3%) were osteoporotic.

**Table 2:** Classification of bone status.

Classification	Total (*n* = 597)	Males (*n* = 286)	Females (*n* = 311)
Normal	245 (41.04%)	58.0%	25.4%
Osteopenic	193 (32.33%)	26.6%	37.6%
Osteoporotic	159 (26.63%)	15.4%	37.0%

**Figure 3:**
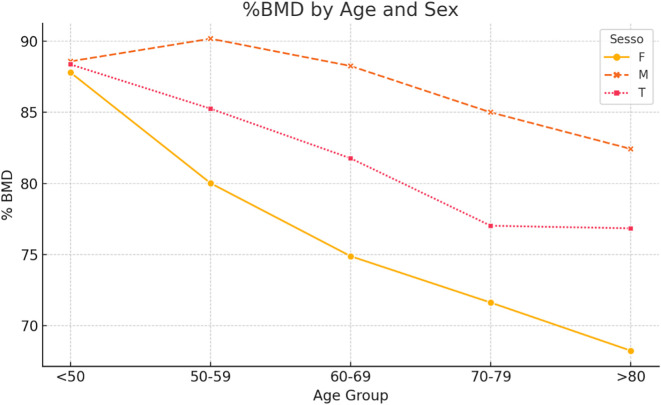
*T*-score by age and sex.

**Figure 4:**
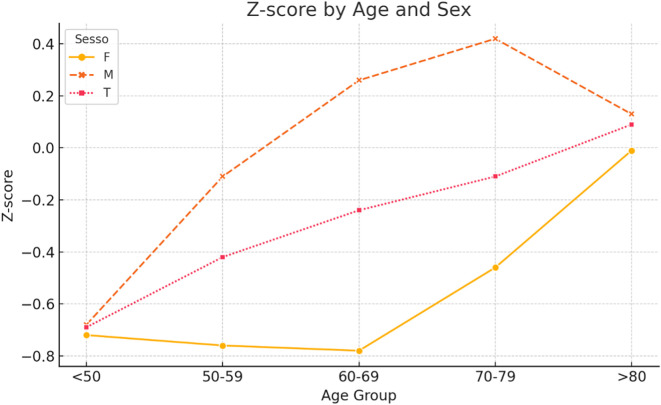
*Z*-score by age and sex.

**Figure 5:**
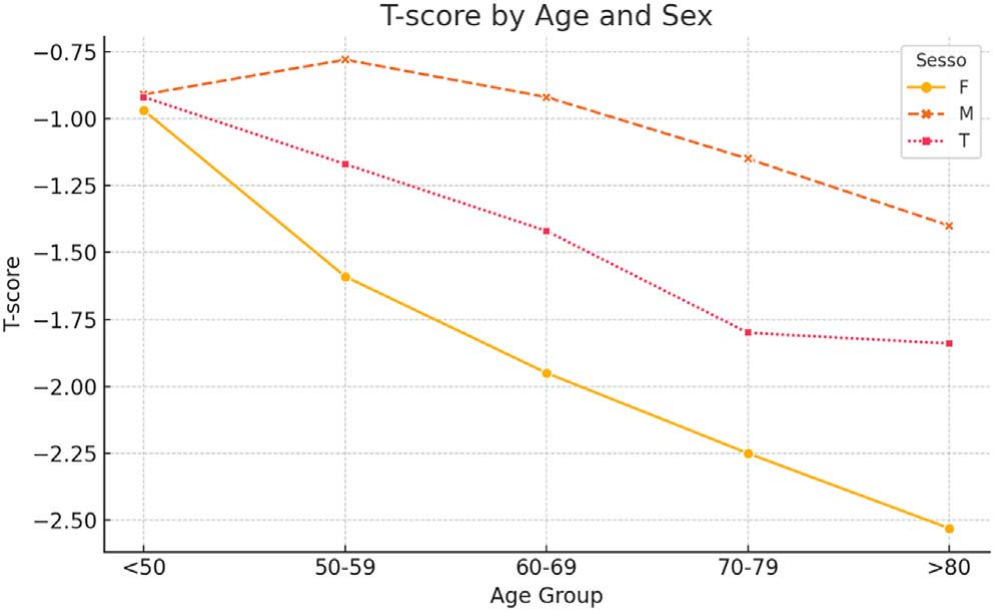
%BMD by age and sex.

When patients were stratified into five age groups (<50, 50–59, 60–69, 70–79, and >80 years), age-related trends were observed (Table 3, Figure 3). Regarding *T*-scores, male patients maintained relatively stable values until the seventh decade, followed by a moderate decline. Female patients demonstrated a sharp decrease in *T*-scores after the age of 60, with mean *T*-scores falling below −2.5 in those over 80 years. *Z*-scores in males remained positive across most age groups, peaking at +0.42 in the 70–79 group, whereas females consistently exhibited negative *Z*-scores across all ages (Table 4, Figure 4). Similarly, %BMD decreased steadily with age in both sexes, with males declining to 82.4% and females to 68.2% in patients aged over 80 years (Table 5, Figure 5).

**Table 3:** *T*-score by age and sex.

Age group	Female	Male	Total
50–59	−1.59	−0.78	−1.17
60–69	−1.95	−0.92	−1.42
70–79	−2.25	−1.15	−1.80
<50	−0.97	−0.91	−0.92
>80	−2.53	−1.40	−1.84

**Table 4:** *Z*-score by age and sex.

Age group	Female	Male	Total
50–59	−0.76	−0.11	−0.42
60–69	−0.78	0.26	−0.24
70–79	−0.46	0.42	−0.11
<50	−0.72	−0.68	−0.69
>80	−0.01	0.13	0.09

**Table 5:** Percentage of BMD by age and sex.

Age group	Female	Male	Total
50–59	80.02	90.18	85.26
60–69	74.89	88.26	81.77
70–79	71.63	85.00	77.03
<50	87.80	88.59	88.37
>80	68.25	82.43	76.85

Multivariate linear regression demonstrated a statistically significant association between age and *Z*-score (*p* < 0.01), with each additional year of age correlating with a 0.02 unit decrease in *Z*-score. Female sex was also an independent predictor of lower *Z*-scores (*p* < 0.01). Categorisation of *Z*-scores using standard thresholds (normal ≥ –1.0, osteopenia between –1.0 and –2.5, and osteoporosis ≤ –2.5) revealed 493 patients with normal bone mass, 139 with osteopenia, and 3 with osteoporosis. Logistic regression confirmed that female patients were 3.77 times more likely to have osteoporosis compared to males (OR = 3.77; 95% CI: 2.45–5.79; *p* < 0.01).

Predicted probabilities further highlighted these differences, with normal BMD present in 68% of females and 86% of males, osteopenia in 30% of females and 11% of males, and osteoporosis in 0.9% of females compared to 0.2% of males. These sex-based differences in bone health distribution were statistically significant (*p* < 0.01).

## Discussion

The main finding of the study is the high prevalence of osteopenia and osteoporosis among patients undergoing robotic-assisted TKA, identified through opportunistic analysis of preoperative CT scans.

This study evaluated the feasibility of opportunistic osteoporosis screening in patients undergoing robotic-assisted TKA by analyzing opportunistic preoperative CT scans using QCT software. Our findings reveal a high prevalence of low BMD in this surgical population, particularly among women and older age groups, underscoring the importance of bone quality assessment in the preoperative setting.

Among the 597 patients analyzed, 58.9% exhibited low bone mass, with 26.6% classified as osteoporotic based on *T*-score values. Notably, 37.0% of female patients were osteoporotic compared to 15.3% of male patients, indicating a significantly higher prevalence in women. These results align with previous studies demonstrating that osteoporosis is more common in women, especially postmenopausal women, due to hormonal changes affecting bone density [14–18]. The progressive decline in BMD with age observed in our cohort further emphasizes the need for targeted screening strategies. In females over 80 years, mean *T*-scores fell below –2.5, and %BMD dropped below 70%, highlighting the increased risk of fragility fractures in this demographic. The multivariate analysis confirmed that both age and sex are significant predictors of low bone mass. Specifically, age was linearly associated with a decrease in *Z*-score, while females were 3.77 times more likely to present with osteoporosis than males. This aligns with the epidemiologic understanding of postmenopausal bone loss and underscores the need for sex-specific risk stratification.

Opportunistic screening using CT scans acquired for TKA planning presents a practical and cost-effective approach to identify patients with undiagnosed osteopenia or osteoporosis. This method leverages existing imaging data without additional radiation exposure or procedural time, facilitating seamless integration into clinical workflows. Previous studies have validated the use of HU measurements from standard CT images as reliable surrogates for BMD, correlating well with DXA values [19–21]. Incorporating bone quality assessment into the preoperative evaluation for TKA can inform surgical decision-making, including implant selection and fixation techniques (cemented vs. cementless, stem extension…). Patients identified with low BMD may benefit from tailored perioperative management and postoperative interventions aimed at improving bone health and reducing the risk of complications such as periprosthetic fractures.

Our results align with, and in some cases, exceed previously reported prevalence rates in the literature. For example, Delsmann et al. reported a prevalence of 17.4% for osteoporosis and 45.9% for osteopenia among elderly patients (≥70 years) undergoing TKA in Germany using DXA-based assessment [22]. Similarly, Daher et al. summarized data indicating that osteoporosis affects 17.4–50% of TKA candidates, with osteopenia affecting up to 64% depending on the population studied and method of assessment [23]. Li et al. reported a comparable prevalence in their Chinese cohort, with osteoporosis found in 30.5% and osteopenia in 44.2% of patients awaiting TKA [24].

Despite the strengths of this study, including a substantial sample size and the use of validated QCT software, certain limitations must be acknowledged. The retrospective design precludes the establishment of causal relationships, and the absence of longitudinal follow-up data limits the ability to assess the impact of low BMD on surgical outcomes. Additionally, while QCT provides accurate BMD measurements, comparisons with DXA, the current gold standard, were not performed in this cohort.

Future prospective studies should aim to validate these findings and explore the predictive value of CT-based bone metrics for postoperative outcomes in TKA patients. Integrating clinical decision tools, such as the Fracture Risk Assessment Tool (FRAX) score, and exploring the role of artificial intelligence in automating HU measurements may further enhance the utility of opportunistic osteoporosis screening in orthopedic practice.

## Conclusion

This study demonstrates the feasibility and clinical utility of opportunistic osteoporosis screening using preoperative CT scans in patients undergoing robotic-assisted TKA. By employing QCT analysis, we identified a high prevalence of low BMD within this surgical population, particularly among elderly female patients.

The integration of QCT-based bone assessment into the preoperative workflow provides a reliable, non-invasive, and cost-effective approach detecting previously undiagnosed osteopenia or osteoporosis. This method requires no additional imaging, radiation exposure, or procedural time, facilitating seamless incorporation into routine clinical practice.

Given the increasing adoption of robotic-assisted systems in arthroplasty and the growing burden of osteoporotic disease, routine opportunistic screening in this setting holds significant translational potential. Prospective studies with longitudinal follow-up are warranted to validate these findings and assess their impact on clinical outcomes and patient care.

## Data Availability

The datasets generated and/or analyzed during the current study are available from the corresponding author on reasonable request.
